# Diminished CD103 (αEβ7) Expression on Resident T Cells from the Female Genital Tract of HIV-Positive Women

**DOI:** 10.20411/pai.v1i2.166

**Published:** 2017-01-16

**Authors:** David C. Moylan, Paul A. Goepfert, Mirjam-Colette Kempf, Michael S. Saag, Holly E. Richter, Jiri Mestecky, Steffanie Sabbaj

**Affiliations:** 1 Departments of Medicine, University of Alabama at Birmingham, Birmingham, Alabama; 2 Department of Microbiology, University of Alabama at Birmingham, Birmingham, Alabama; 3 School of Nursing and Department of Health Behavior, University of Alabama at Birmingham, Birmingham, Alabama; 4 Obstetrics and Gynecology, University of Alabama at Birmingham, Birmingham, Alabama

**Keywords:** Tissue Resident Memory T cells, female genital tract, HIV-1, CD103, mucosal immunity, CD8^+^ T cells, CD4 T cells

## Abstract

**Background:**

Tissue resident memory T cells (TrM) provide an enhanced response against infection at mucosal surfaces, yet their function has not been extensively studied in humans, including the female genital tract (FGT).

**Methods:**

Using polychromatic flow cytometry, we studied TrM cells, defined as CD62L-CCR7-CD103^+^CD69^+^ CD4^+^ and CD8^+^ T cells in mucosa-derived T cells from healthy and HIV-positive women.

**Results:**

We demonstrate that TrM are present in the FGT of healthy and HIV-positive women. The expression of the mucosal retention receptor, CD103, from HIV-positive women was reduced compared to healthy women and was lowest in women with CD4 counts < 500 cells/mm^3^. Furthermore, CD103 expression on mucosa-derived CD8^+^ T cells correlated with antigen-specific IFN-γ production by mucosal CD4^+^ T cells and was inversely correlated with T-bet from CD8^+^CD103^+^ mucosa-derived T cells.

**Conclusions:**

These data suggest that CD4^+^ T cells, known to be impaired during HIV-1 infection and necessary for the expression of CD103 in murine models, may play a role in the expression of CD103 on resident T cells from the human FGT.

**STANDFIRST**

The expression of CD103 on T cells in the human FGT likely requires CD4^+^ T cells.

## INTRODUCTION

There is a large gap in our understanding of the role cell-mediated immunity plays in controlling infections within mucosal tissues and how local T-cell memory is established after infection between animal models and humans. Furthermore, there is limited information available from studies of the female genital tract (FGT) as compared to the gastrointestinal tract and other mucosal tissues. Data obtained from other mucosal sites may not be applicable to the FGT because the FGT must balance the induction of a protective immune response with the ability to preserve the developing fetus.

The ability of memory T cells to migrate into mucosal tissues allows these cells to gain access to the site of active infection. The expression of αEβ7 (mucosal retention receptor, CD103), and the downregulation of CCR7 and CD62L (lymph node homing receptors) are known mechanisms leading to the maintenance of cells in mucosal tissue such as the lung, gut, and genital tract [1–5]. It is well established that HIV-specific CD8^+^ T cells isolated from peripheral blood mononuclear cells (PBMC) express distinct memory phenotypes compared to other virus-specific T cells (reviewed in [6]) but information on mucosa-derived cells is lacking. The peripheral blood and mucosal compartments have been shown to contain distinct memory T-cell subsets that exhibit characteristic homing, differentiation, activation, polyfunctional, and proliferation capabilities [4, 7–10], strongly suggesting unique functional abilities depending on where these T cells are located. This is turn supports the view that the site of migration influences the phenotype and function of these cells [11–14]. In addition, different memory T-cell subsets have specific abilities to control viral infections. For example, central memory T cells (Tcm) are long-lived memory cells that control infection after reencountering the pathogen and expanding, differentiating and trafficking to the appropriate compartment. On the other hand, effector memory T cells (Tem), are short-lived, differentiated, and located at the site of infection, and are capable of controlling infections much more rapidly [14, 15]. In fact, studies with rhesus macaques demonstrated that a vaccine using CMV as the vector elicited Tem and was capable of early control of SIV [16]; this was not seen with traditional CD8^+^ T-cell vaccines that elicit predominantly Tcm responses [17]. Studies in mice [11, 12, 18–25] have revealed a relatively new type of memory T cell, the tissue resident memory T cell (TrM). Recently, TrM have been studied in multiple human mucosal tissues, except for the FGT, obtained from organ donors [26]. These cells are maintained in non-lymphoid tissue and are thought to be stationary. Conflicting data in animal models as to whether TrM cells can be retained in tissues such as the skin, vagina, and intestinal tract in the absence or presence of antigen, suggest that these differences may vary depending on location and type of infection. Most importantly, these cells are not present in the peripheral blood and can only be studied by direct examination of non-lymphoid tissue. TrM cells have been proposed, together with Tem, to be the only cells capable of providing immediate protective immunity in tissues. However, phenotypically defined (CD62L-CCR7-CD103^+^CD69^+^) resident memory T cells [27] have not been described in the human FGT.

In mice, there is ample information available concerning the mechanism and signals T cells undergo to become protective TrM in the different tissues in which they reside. In this process CD4^+^ T cells have been implicated in the migration of effector CD8^+^ T cells into the FGT [28], but not the skin [21]. More recently, it has been shown that CD4^+^ T cells are needed to guide the formation of CD103^+^ lung resident memory CD8^+^ T cells [29]. In addition, the downregulation of the transcription factor, T-bet, has been shown to be necessary for the expression of CD103 [29, 30]. Clearly these types of studies are very difficult to perform in humans. However, by studying TrM in HIV-positive women—individuals with dysregulated CD4^+^ T cells—we can determine whether the data obtained in murine models translate to humans.

In this report, we extend our studies using menstrual blood (MB) as a surrogate mucosal tissue [31] along with tissues from the FGT to demonstrate that TrM CD8^+^ T cells are present in the FGT of healthy as well as HIV-positive women. We were also able to demonstrate the diminished expression of the homing retention receptor CD103 on mucosa-derived T cells in these HIV-positive women. Our data suggest that, in humans, the expression of CD103 on mucosa-derived T cells is also dependent on local CD4^+^ T cell help.

## METHODS

### Subjects

Menstruating healthy women (N = 13) and HIV-positive women (N = 27, Table 1) were recruited from the University of Alabama at Birmingham's (UAB) 1917 Clinic, the Alabama Vaccine Research Clinic, and the Women's Interagency HIV Study (WIHS) cohort at UAB to donate menstrual blood and peripheral blood. The ages for the healthy women ranged between 20-43 years, with the median age 36 years. With the exception of one Caucasian woman, all volunteers were African American. Women enrolled in these studies had no active STI. Menstrual cups were provided to volunteers prior to menstruation with instructions on how to use them. Contents of menstrual cups were decanted into 50 ml tubes containing amphotericin B (0.5 mg/ml), penicillin/streptomycin (100 U/ml), and gentamicin (100 mg/ml) plus acid-citrate-dextrose solution (ACD) as the anticoagulant (5 ml/tube). The antimicrobial and anticoagulant volumes added to the 50 ml tubes were determined assuming 25 ml of menstrual blood/tube. Samples were kept at room temperature (RT) and brought to the laboratory within 6 hours of collection. In addition, vaginal (N = 36) and endometrial (N = 8) tissue samples, derived as anonymous remnant surgical material, were obtained from otherwise healthy women undergoing reconstructive pelvic surgery or a hysterectomy. Written informed consent was obtained from all women who participated in this study. The Institutional Review Board of the University of Alabama at Birmingham approved the study.

**Table 1.** Clinical information and demographics of HIV-infected women

Volunteer#	Age	Race/Ethnicity	ART^a^	Plasma VL^b^ (copies/ml)	Absolute CD4 (cells/mm^3^)	CD4 Nadir (cells/mm^3^)	ART Length years
1C-301	31	AA	yes	<20	809	389	9
1C-302	32	AA	yes	<20	866	616	2
1C-303	31	AA	yes	<20	763	486	6
1C-304	48	AA	yes	<20	661	154	4
1C-305	34	AA	yes	<20	623	301	7
1C-306	32	AA	yes	<20	501	435	5
1C-307	38	C	yes	<20	645	262	3
1C-308	37	AA	yes	<20	1019	431	6
1C-309	41	AA	no	<20	1038	851	-
1C-310	49	AA	no	<20	992	729	-
1C-311	46	AA	yes	<20	923	356	7
1C-312	39	AA	yes	122	313	220	5
1C-313	38	AA	yes	<20	849	460	5
1A-101	35	AA	yes	<20	426	23	6
1A-102	35	AA	yes	<20	286	49	18
1A-103	40	AA	yes	92	443	39	4
1A-104	35	AA	yes	35	1578	575	5
1A-105	37	AA	yes	445	696	363	1^c^
WIHS 1	30	AA	yes	<20	616	616	1
WIHS 2	46	AA	yes	<20	399	154	8
WIHS 3	39	AA	no	<20	1048	974	**-**
WIHS 4	36	AA	no	9369	280	276	**-**
WIHS 5	45	AA	yes	10 700	169	44	3
WIHS 6	37	AA	yes	<20	349	220	3
WIHS 7	43	AA	yes	3720	803	416	5
WIHS 8	35	AA	yes	<20	1096	460	3
WIHS 9	35	AA	yes	<20	433	275	2

a Antiretroviral therapy

b Viral load

c month

### Isolation of menstrual blood cells

MB was obtained from women on day 1 and/or 2 of the menstrual cycle. Briefly, MB was diluted 1:2 in PBS, without Ca^++^/Mg^++^, layered onto Ficoll-Paque PLUS (GE Healthcare, Piscataway, NJ) and centrifuged at 800 x g for 25 min. at RT as previously described [31]. Interface containing buffy coat was removed; PBS was added and spun down at 400 x g for 10 min twice. If the cell pellet contained RBC, it was spun down, the supernatant discarded, and the pellet resuspended. Then 5mL cold ammonium-chloride-potassium buffer (ACK) was added to the pellet for 5 min. Cell lysis was stopped by filling the tube with PBS and centrifuging at 400 x g for 10 min. Super-natant was discarded. If the pellet still appeared to have RBC, this procedure was repeated up to 2 more times. Cell pellets were resuspended in media. If the sample contained too many particulates, cell suspension was passed through a 70 μM nylon cell strainer (BD Falcon, Franklin Lakes, NJ) to remove residual tissue. PBMC were obtained by standard density centrifugation. Cells were counted and used for surface staining and/or intracellular cytokine assays.

### Isolation and purification of lymphocytes from tissue

Tissue segments were obtained as either vaginal or endometrial surgical samples after pelvic reconstructive surgery. Lymphocytes were isolated by dissecting and mincing tissue with a scalpel into the smallest possible fragments. Fragments were transferred to 50 ml conical tubes containing 30 ml of warm 0.05% collagenase (Sigma type II collagenase #C6885) in 7.5% FBS RPMI media and incubated for 15 min at 37°C. Partially digested tissue was mechanically disrupted 2x using a 16g blunt needle attached to a 20ml syringe. Tissue was incubated for an additional 15 min at 37°C. Cell suspension was passed through a 40 μM nylon cell strainer (BD Falcon, Franklin Lakes, NJ) and RPMI + 15% FBS (R15) was added to quench enzyme activity. Cell suspension was pelleted 2x at 300 x g for 10 min at RT. After the second wash, the cell pellet was resuspended in 5 ml of R15, and placed at 37°C with a loose cap. Collagenase digestion was repeated 2x. All 3 pellet fractions were combined and resuspended in 50 ml R15 and pelleted at 300 x g for 10 min. Supernatant was discarded and the remaining cell suspension was resuspended in 1-3 ml of RPMI containing 10% AB serum. Cells were counted and used for the various assays.

### Surface marker staining

Phenotypic characterization of menstrual blood cells (MBC), mononuclear cells isolated from tissue, and PBMC employed cell-surface markers CD3-Alexa780 (eBioscience, San Diego, CA), CD8-V500, CD45-PEcy7, CD62L-FITC, CD69-APC, CD103-PE (αEβ7), CD197-PercpCy5.5 (CCR7), CD4-Qdot655 (ThermoFisher, Waltham, MA), and fluorescent LIVE/DEAD Fixable Blue Dead Cell Stain (Molecular probes, Invitrogen, CA). Stained cells were acquired using a BD Calibur flow cytometer (Becton Dickinson [BD], San Jose, CA) and analyzed with FlowJo Version 9.9 software for Mac (TreeStar, San Carlos, CA). We ran at least 100,000 gated lymphocytes for each stained specimen. Fluorescence minus one (FMO) on cells stimulated with staphylococcus enterotoxin B (SEB) was used to set gates for the TrM cells (CD62L-, CCR7-, CD103^+^, CD69^+^). All antibodies were obtained from BD Biosciences, San Jose, California unless otherwise noted.

### Antigens

Pools of overlapping HIV peptides (15-mers overlapping by 11, NIH AIDS repository) from the following proteins: Gag, Pol, Nef, and Env. PDBu/ionomycin (phorbol-12,13 dibutyrate) and/or SEB (Sigma-Aldrich, St. Louis, MO) were used as positive controls and unstimulated cells as a negative control.

### Intracellular cytokine staining

Intracellular cytokine staining was performed as previously described [31, 32]. MBC, PBMC, and mononuclear cells isolated from tissue were resuspended in RPMI containing 10% human AB serum, and co-stimulatory monoclonal antibodies (anti-CD28 and anti-CD49d, each at 1 μg/ ml) were added to each tube along with 50 U/ml of Benzonase (Novagen, Madison, WI). Cells were pulsed with the appropriate antigen followed by the addition of 10 μg/ml Monensin (Golgistop, BD Biosciences, San Jose, CA). Cells were then incubated at 37°C, 5% CO_2_, for 6 hours, and placed at 4°C overnight. The following day cells were labeled with a fluorescent LIVE/DEAD fixable Blue Dead Cell Stain (Molecular probes, Invitrogen, CA). The surface phenotype of samples was determined by first staining with CCR7 and placing in a 37°C water bath for 20 min in the dark. This was followed by staining with the remaining surface antibodies for 20 min at RT in the dark. Cells were washed in PBS, followed by permeabilization with the Cytofix/cytoperm reagent (BD, San Jose, CA) for 20 min at RT in the dark. Intracellular cytokine staining followed using anti-IFN-γ-Alexa700 conjugated antibody. Using a second panel of antibodies, T-bet conjugated to Alexa Fluor488 was also measured. At least 100,000 lymphocytes were acquired from each sample using a BD LSR II flow cytometer (Becton Dickinson [10], San Jose, CA). Data were analyzed using FlowJo Version 9.9 software (TreeStar, San Carlos, CA). Lymphocytes were analyzed based on forward and side scatter profiles for PBMC and by gating on CD45 for MB and tissues followed by the exclusion of dead cells. Cytokines produced were measured using the CD3^+^CD4^+^ gates relative to the media control values and these gates were applied to all samples with different antigens from the same individual. Surface staining was determined using FMOs as above. All antibodies were obtained from BD Biosciences, San Jose, California unless otherwise noted. We used Fisher's exact test to compare the number of cytokine-producing cells between the antigen-stimulated and unstimulated (media alone) samples to determine whether a response is considered positive. A *P* < 0.05 was used for CD4^+^ responses from MBC. To be considered a positive response the frequency also had to be > 0.05%.

### Statistics

Statistical analyses were performed using the non-parametric Wilcoxon Signed Rank test for paired samples; otherwise the Mann-Whitney U test was used. Analyses were done with Graph-pad Prism software 5.0 for Mac. Differences were considered to be significant on the basis of 95% confidence intervals (*P* < 0.05).

## RESULTS

### Tissue Resident Memory T cells (TrM) in the female genital tract

**Figure 1.**
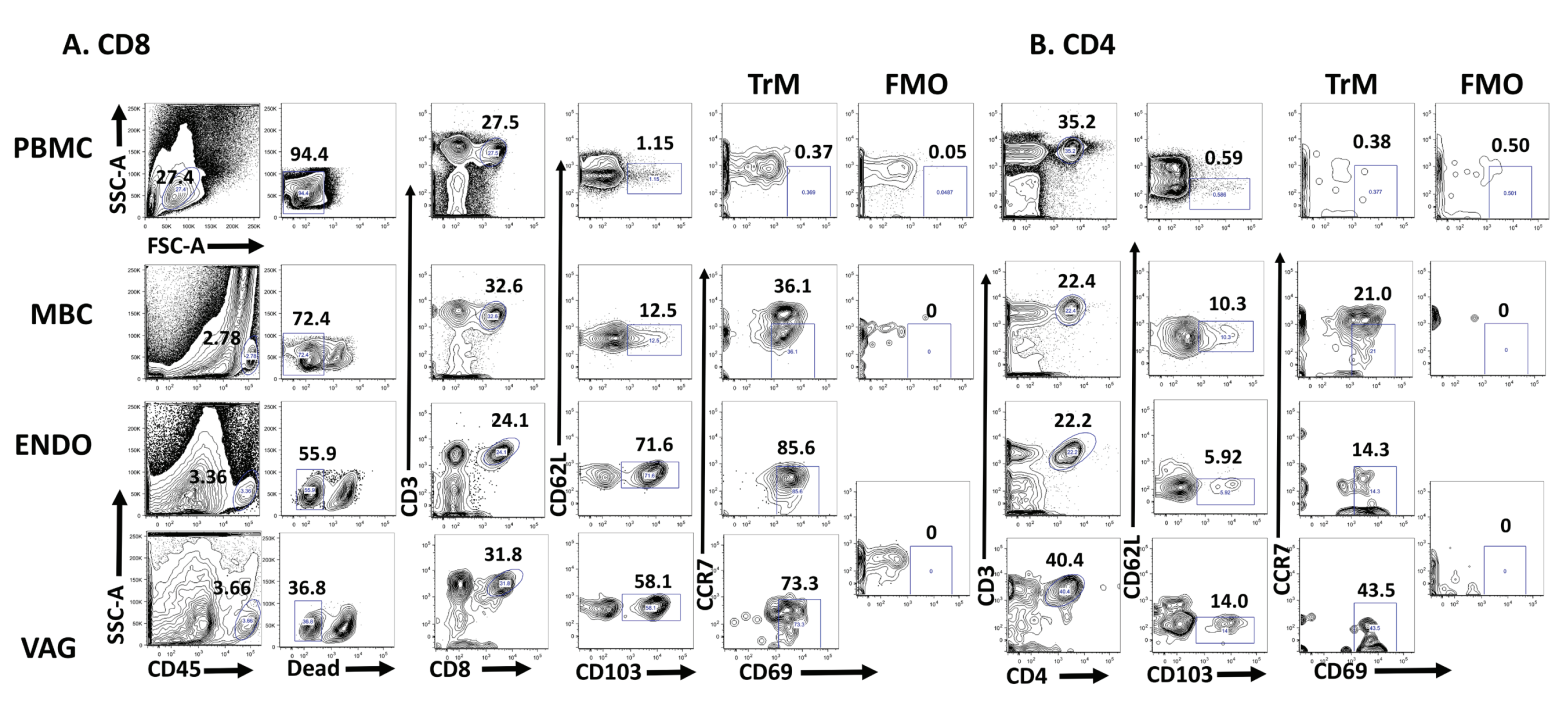
**Sample gating strategy.** (A) The percent CD3^+^CD8^+^ and (B) CD3^+^CD4^+^ Tissue Resident T cells (CD62L-CD103^+^CCR7-CD69^+^) is shown. The flow plots represent TrM cells present in paired PBMC and MBC from an HIV-positive woman and endometrial (ENDO) and vaginal tissue from an anonymous remnant sample. Fluorescence minus one (FMO) is set individually for PBMC, MBC, and ENDO/VAG (which share the same FMO, because they are from the same donor). The percent of the gated population is shown above the gate.

**Figure 2.**
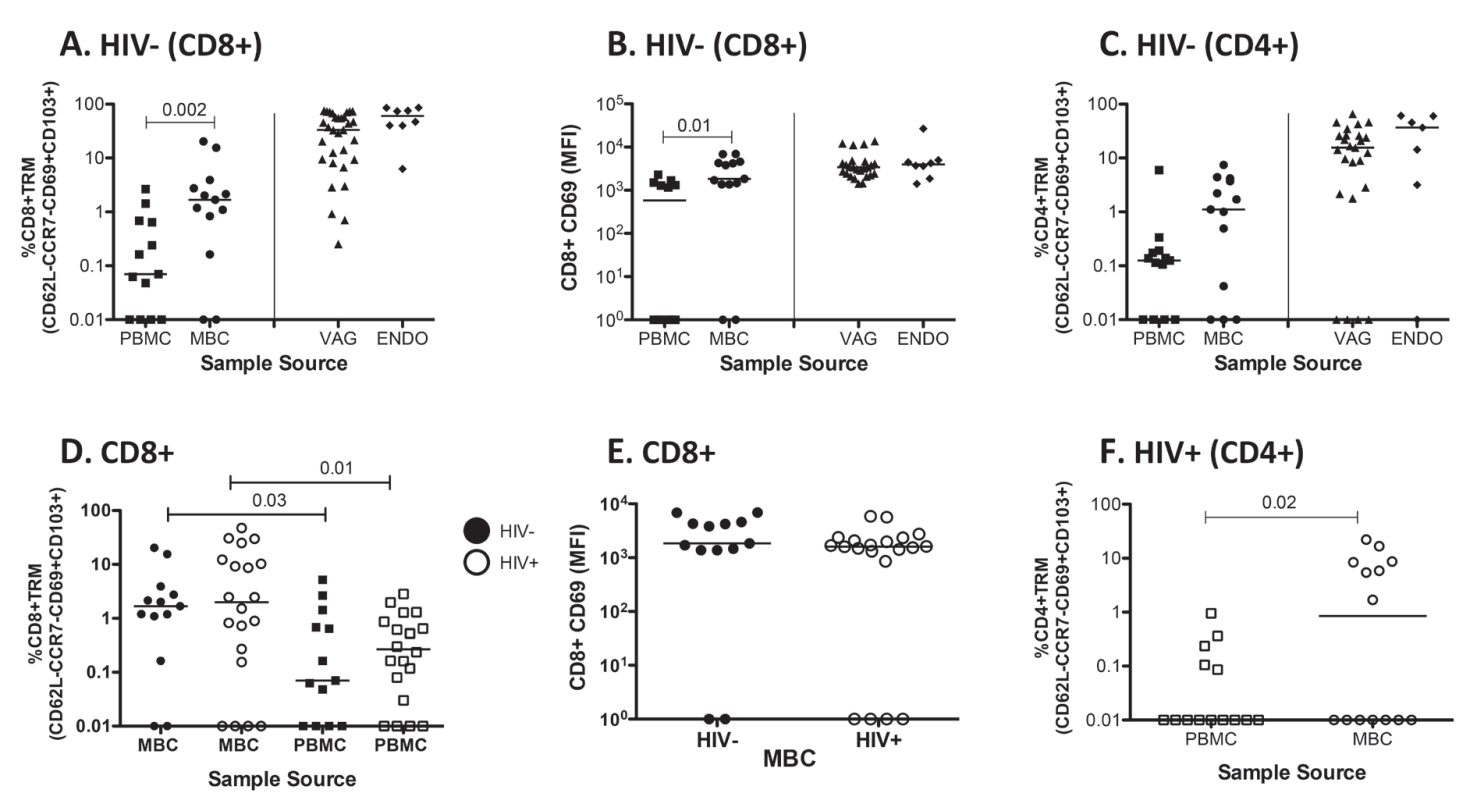
**Tissue Resident T cells are present in the FGT.** (A) The percentage of CD3^+^CD8^+^ TrM (CD62LCD103^+^CCR7-CD69^+^) cells present in paired PBMC and MBC from HIV-negative women and from vaginal (VAG) and endometrial (ENDO) tissue from anonymous remnant samples. (B) Mean Fluorescence Intensity (MFI) of CD8^+^CD69^+^ T cells for paired PBMC and MBC from HIV negative women and from VAG and ENDO tissue from anonymous remnant samples. (C) The percentage of CD3^+^CD4^+^ TrM cells present in paired PBMC and MBC from HIV-negative women and VAG and ENDO tissue from anonymous remnant samples. (D) The percent of CD3^+^CD8^+^ TrM present in paired MBC and PBMC between HIV-negative and positive women. (E) CD69 MFI between CD8^+^CD69^+^ TrM from MB cells from HIV negative and positive women. (F) The percentage of CD3^+^CD4^+^ TrM cells present in paired PBMC and MBC from HIV positive women. Fluorescence minus one (FMO) is set individually for PBMC and MBC. Horizontal line represents median value. Statistically significant differences (*P* < 0.05) were obtained using the Wilcoxon Rank test for paired samples and for unpaired we used the Mann Whitney test. Although not noted in the graph, differences were also detected between PBMC and MBC and tissues (VAG and ENDO).

Several studies have suggested the presence of TrM in the FGT of humans [33, 34]. To determine whether tissue-resident memory T cells defined as CD62L-CCR7-CD103^+^CD69^+^ are established in the FGT of humans, we used cells obtained from MB and paired PBMC from HIV-positive and healthy women. In addition, vaginal (VAG) and endometrial (ENDO) samples from anonymous remnant tissue were used as a source of T cells. The gating strategy we used to detect CD8^+^ (Figure 1A) and CD4^+^ (Figure 1B) TrM cells is depicted. Fluorescence minus one (FMO) was used to determine where to place the CD69 gate. In Figure 2A we compare the presence of TrM in PBMC and MBC from HIV-negative women and from VAG and ENDO tissue from anonymous remnant samples. By gating on live CD3^+^CD8^+^CD62L-CD103^+^CCR7-CD69^+^ T cells, we demonstrated that TrM CD8^+^ T cells are present in menstrual blood cells (MBC, median 1.7%), vaginal (VAG, median 29%) and endometrial (ENDO, median 6%) tissue but diminished in PBMC (median 0.07%) (Figures 1 and 2A).

Moreover, when we compare the frequency of TrM between MBC from HIV-negative (closed circles) and HIV-positive (open circles) samples we detect no differences (Figure 2D). TrM are also enriched in MBC from HIV-positive samples (median 2.5%) when compared to paired PBMC (median 0.25%) (Figure 2D). When we compared the MFI of CD8^+^CD69^+^ T cells from PBMC and MBC from the HIV-negative women, we also detected significant differences between PBMC and MBC (Figure 2B) in the expression of CD69. However, the MFI of CD8^+^CD69^+^ on T cells isolated from MB was not different between HIV-negative and HIV-positive samples (Figure 2E). We then analyzed whether CD3^+^CD4^+^ TrM were present in the FGT of HIV-negative women by using MBC (median 1.1%) and tissue (VAG, median 15.5%, and ENDO, median 36.8%) (Figure 2C). Our data demonstrate that CD4^+^ TrM are also present in the FGT when compared to PBMC (median 0.11%). When we analyzed CD4^+^ TrM in HIV-seropositive women, these were also detected (Figure 2F). Just as with CD3^+^CD8^+^ TrM, we observed no differences in the percentage of TrM or the expression of CD69 (MFI) between HIV-positive and negative women when we analyze CD3^+^CD4^+^ T cells isolated from MB (data not shown).

### Reduced CD103 expression on T cells derived from the female genital tract of HIV-positive women

**Figure 3.**
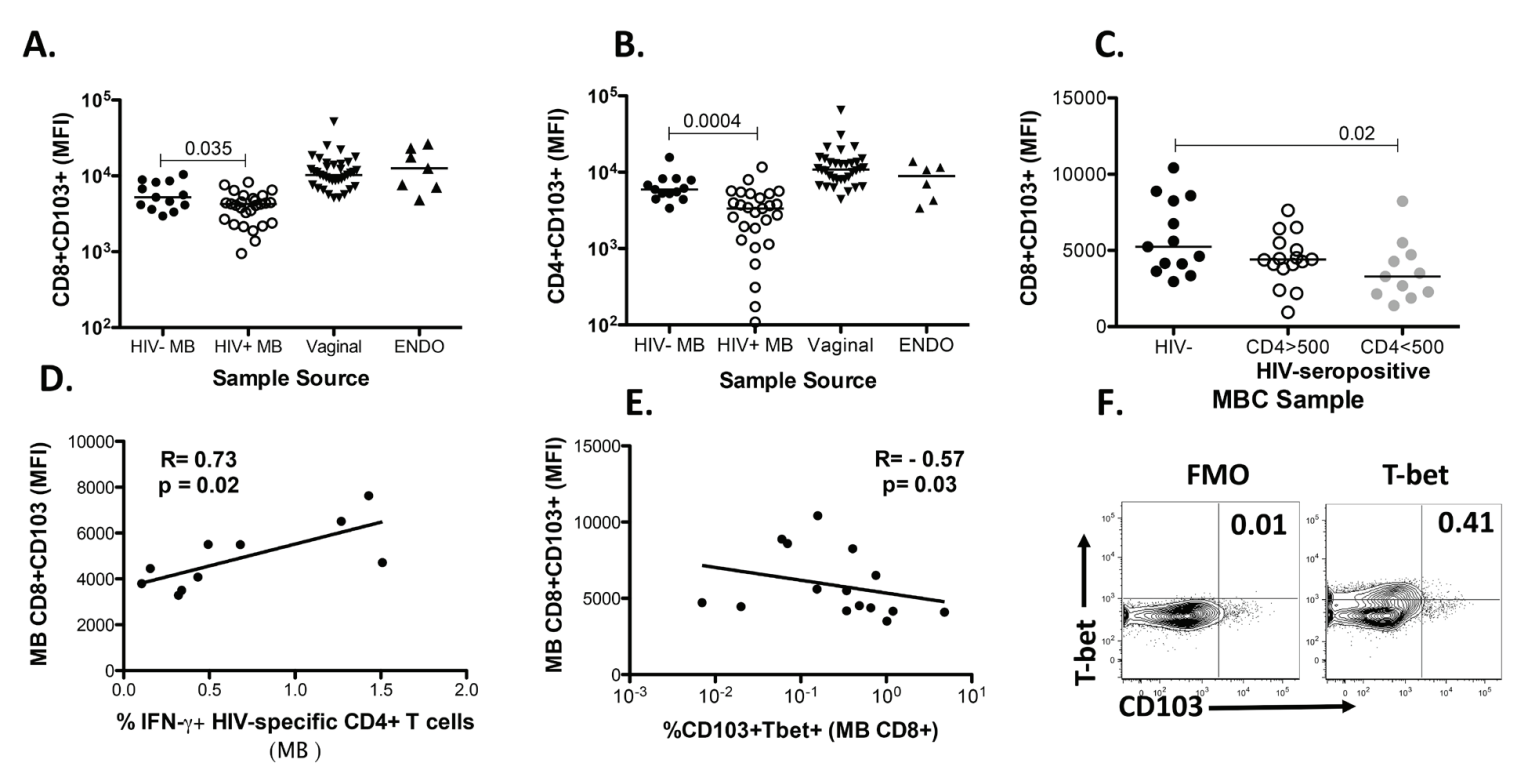
**Reduction in the expression of CD103 in MB from HIV-positive women.** (A) The mean fluorescence intensity (MFI) of CD103 expression on CD3^+^CD8^+^ T cells. (B) The MFI of CD103 expression on CD3^+^CD4^+^ T cells. T cells were isolated from MB from HIV-positive and negative women and vaginal and endometrial samples from anonymous samples. (C) The MFI of CD103 expression on CD3^+^CD8^+^ T cells isolated from MB from HIV-negative and HIV-positive women stratified by absolute CD4 T cell number (cells/mm^3^) (D) Correlation between CD103 expression (MFI) on CD103^+^CD3^+^CD8^+^ T cells isolated from menstrual blood of HIV-positive women and the production of IFN-γ from CD4^+^CD3^+^ T cells isolated from menstrual blood of HIV-positive women specific for HIV. The IFN-γ production from CD4^+^ T cells from all HIV antigens demonstrating a positive response was added for each individual. (E) Correlation between CD103 expression (MFI) on CD103^+^CD3^+^CD8^+^ T cells isolated from menstrual blood and the percent of T-bet^+^ CD8^+^CD3^+^CD103^+^ T cells isolated from menstrual blood. (F) Sample gating strategy for T-bet from CD8^+^ T cells isolated from MB from an HIV-infected sample. Horizontal line represents median value. Statistically significant differences (*P* < 0.05) were obtained using the Wilcoxon Rank test for paired samples and the Mann Whitney test for unpaired samples. Although not noted in the graph, differences were also detected between MBC and tissues (VAG and ENDO). Correlations were determined using the Spearman correlation.

Recent studies in mice observed that homing of TrM cells is impaired when CD4^+^ T cell help is lacking due to the inability to express the mucosal retention receptor CD103 [29]. This impairment in CD103 expression is dependent on the production of IFN-γ by CD4^+^ T cells and could be rescued with the reduction of T-bet expression on CD8^+^ TrM. Consequently, we wanted to determine whether CD4 help plays a similar role in humans and analyzed the expression of CD103 on MBC from HIV-positive women (our impaired CD4 T cell help group) versus healthy women. We demonstrated that the expression of CD103 was reduced on CD103^+^CD3^+^CD8^+^ and CD103^+^CD3^+^CD4^+^ (Figures 3A and 3B) T cells isolated from HIV-positive menstrual blood (HIV+ MB) compared to negative samples (HIV− MB). We further demonstrate that if we stratify the HIV-positive women by their absolute CD4 count, differences in the expression of CD103 are observed between CD103^+^CD8^+^ T cells isolated from MB from the HIV-negative women (closed black circles) when compared to CD103^+^CD8^+^ T cells from HIV-positive women with absolute CD4 counts less than 500 cells/mm^3^ (gray circles) (Figure 3C). These data also indicate that the differences in the expression of CD103 on T cells between HIV-infected and uninfected women is most likely driven by the samples from the HIV-positive women with CD4 < 500 cells/mm^3^. If we stratify the samples based on whether the women were viremic (VL > 20) or aviremic, there is a trend (*P* = 0.09) for higher CD103 expression in the aviremic group (data not shown). Although the clinical absolute or percent of CD4 did not correlate with the expression of CD103 (data not shown), the expression of CD103 on CD103^+^CD8^+^ T cells isolated from the MB of HIV-positive women correlated with HIV-specific IFN-γ production from CD4^+^ T cells isolated from the MB from these women (Figure 3D). Lastly, the expression of CD103 was inversely correlated with T-bet on CD8^+^ T cells isolated from MB (Figure 3E), suggesting that T-bet downregulation is necessary for the expression of CD103, as shown in murine studies [29]. A sample gating strategy for T-bet is shown in Figure 3F. It is worth noting that most of the HIV-positive women were on cART and many had CD4 T-cell numbers within the normal range (Table 1).

## DISCUSSION

It has been shown repeatedly that TrM play an essential role in the containment of non-lymphoid infections of the skin, lung, and gastrointestinal and genital tracts in murine models [19, 21, 24, 29, 35–38]. In humans only a few studies have been conducted, primarily using skin TrM [34, 39, 40]. Recently, studies examining CD8^+^ T cells in the FGT [33, 34], were carried out whereby it was demonstrated that HSV-2-specific T cells were present in the FGT. However, phenotypic characterization of TrM using LN homing receptors (CCR7 and CD62L) and the mucosal retention receptor CD103 along with CD69 expression were not performed, in order for parallels to murine studies to be made. Analyses of human FGT are challenging due to the need for large tissue samples to obtain sufficient numbers of cells. Therefore, remnant tissues are used instead of small biopsies. Although remnant tissues are useful for many immunologic evaluations, antigen-specific studies are precluded and therefore associations with viral control, for example, are not possible. In addition, the inability to perform challenge experiments, to use knockouts, or to treat with depleting antibodies limits the kinds of studies possible in humans. Despite these drawbacks, in this report we demonstrated that, phenotypically, CD62L-CCR7-CD103^+^CD69^+^ TrM CD8^+^ T cells are present in the FGT of both healthy and HIV-positive women. We also established the presence of CD3^+^CD4^+^ TrM cells (Figures 2C and 2F) as has been described previously in animal models [36, 41–43] and recently in humans [44]. Lastly, we demonstrated that the expression of the retention receptor CD103 is diminished in HIV-positive women.

Our data support the results of others [28] whereby CD4^+^ T cells were required for the migration of effector CD8^+^ T cells into the FGT. We demonstrated that the expression of CD103 is reduced on MB T cells isolated from HIV-positive women (Figures 3A and 3B) and that this reduction is most pronounced on CD8 T cells from HIV-infected women with reduced CD4 T cell numbers (Figure 3C). These results parallel data from Laidlaw *et al*. (2014), which demonstrate that functional CD4^+^ T cells are needed in the formation of lung CD103^+^CD8^+^ T cells. Together our data suggest that, in humans too, functional CD4^+^ T cells are necessary for the formation of CD8^+^ tissue resident T cells.

However, despite all of these associations, we were unable to correlate either absolute CD4 or percent of CD4 values obtained from clinical laboratory results with the presence of TrM in HIV-positive women. Our data suggest that despite the normal range of CD4 T cells, HIV-positive women most likely have impaired CD4^+^ T cells [45, 46] that are incapable of helping in the establishment of resident CD8^+^CD103^+^ T cells in the FGT. To this end we demonstrated that the expression of CD103 on CD8^+^ T cells isolated from MB correlates with HIV-specific CD4^+^ T cells present in MB (Figure 3D) and is inversely correlated with the transcription factor T-bet, known to be downregulated during CD103 expression in mice (Figure 3E).

**Supplemental Figure 1.**
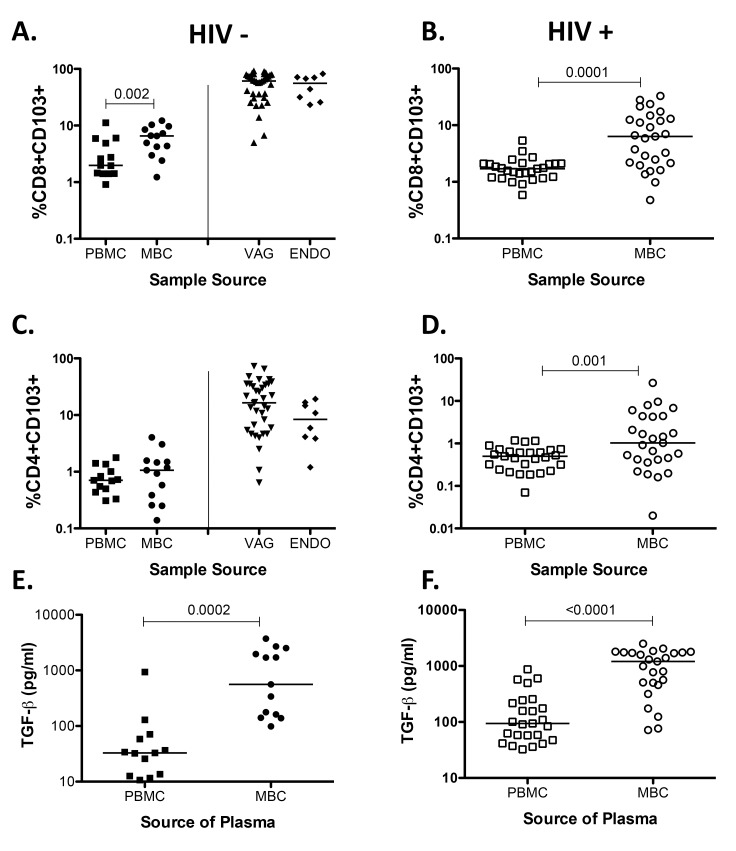
**Percent of CD103 and TGF-β concentration from HIV-seronegative samples (closed symbols) and HIV-seropositive samples (open symbols).** (A) %CD8^+^CD103^+^. (B) %CD8^+^CD103^+^. (C) %CD4^+^CD103^+^ (D) %CD4^+^CD103^+^ (E) TGF-β concentration in plasma from PBMC and MBC from HIV-seronegative samples. (F) TGF-β concentration in plasma from PBMC and MBC from HIV-seropositive samples. Horizontal line represents median value. Statistically significant differences (*P* < 0.05) were obtained using the Wilcoxon Rank test for paired samples. Although not noted in the graph, differences were also detected between MBC and tissues (VAG and ENDO).

When we analyzed the data using percent of CD103 instead of the MFI of CD103 expression on T cells we noted that differences between PBMC and MBC remain (Supplemental Figures 1A, 1B, and 1D), but differences between HIV-infected and uninfected samples were not seen (data not shown). These data suggest that the decrease in the expression of CD103 is due to a reduction in the expression of CD103 and not to lower CD103^+^ T cell numbers. TGF-β has been shown to be necessary for the expression of CD103 in murine models [47, 48] Therefore we measured the concentration of TGF-β1 in plasma obtained from menstrual blood, and noted that it is significantly elevated compared to plasma obtained from peripheral blood (Supplemental Figures 1E and 1F), in line with a previous study showing the presence of TGF-β1 using cervicovaginal lavage samples [49] and supporting data demonstrating that it is produced in the human endometrium [50]. However, differences between HIV-infected and healthy plasma samples were not detected (data not shown).

New evidence in murine studies suggests that TrM should be defined as resident cells in tissue that do not recirculate, because there appears to be substantial phenotypic heterogeneity in terms of surface expression of CD103 and CD69 on these cells [51, 52]. This observation should be taken into account when analyzing cells from human tissue, as single positive cells may also include resident memory cells (eg, CD103^+^CD69− or CD103−CD69^+^). This discrepancy may partially explain why no differences are observed between HIV-positive and negative samples when CD103^+^CD69^+^ cells are analyzed.

Although it is clear that MBC are not an exact representation of tissue-derived cells from the FGT and that TrM are lower in frequency in MB than in tissue, MB is still a useful substitute when studying cell-mediated immunity in the FGT. Our data using MB corroborate data from others in which a reduction in CD103^+^ T cells was observed between HIV-positive and HIV-negative individuals in other mucosal compartments such as the gastrointestinal tract [53] and breast milk [54], suggesting this dysfunction extends to various mucosal compartments.

Because most women in this report were receiving cART we were unable to determine whether TrM in the FGT play a role in HIV control. Despite this fact, the results generated demonstrate that TrM T cells are present in the FGT of women and that HIV infection impairs their function and/or localization. Moreover, we were unable to discern the role antigen plays in the generation or maintenance of TrM in the FGT. Further experiments aimed at determining antigen role in the maintenance and development of TrM need to be carried out.

Given that at the time of menstruation all hormones involved in the menstrual cycle are at their lowest levels, MB-derived cells should not be influenced by hormonal levels and comparisons between women should also not be influenced by differences in hormonal concentrations. Therefore, MB can now be added as one of the various sources of mucosal sampling available to study cell-mediated immunity in the FGT.

Understanding resident memory T-cell function in human tissues may enable our understanding of the type of cells needed to be generated in preventative vaccines for protection from pathogens infecting the mucosal surfaces of the FGT. Finally, this information adds to the body of knowledge of the human mucosal immune system.
